# Little evidence of a road‐effect zone for nocturnal, flying insects

**DOI:** 10.1002/ece3.4609

**Published:** 2018-12-27

**Authors:** Manisha Bhardwaj, Kylie Soanes, José J. Lahoz‐Monfort, Linda F. Lumsden, Rodney van der Ree

**Affiliations:** ^1^ School of BioSciences University of Melbourne Parkville Victoria Australia; ^2^ School of Ecosystem and Forest Sciences University of Melbourne Parkville Victoria Australia; ^3^ Department of Environment, Land, Water and Planning Arthur Rylah Institute for Environmental Research Heidelberg Victoria Australia; ^4^ Ecology and Infrastructure International Wantirna Victoria Australia

**Keywords:** avoidance, biomass, habitat degradation, impacts of roads, invertebrates, orthopteran, predator–prey interactions, road ecology

## Abstract

Roads and traffic may be contributing to global declines of insect populations. The ecological effects of roads often extend far into the surrounding habitat, over a distance known as the road‐effect zone. The quality of habitat in the road‐effect zone is generally degraded (e.g., due to edge effects, noise, light, and chemical pollution) and can be reflected in species presence, abundance, or demographic parameters. Road‐effect zones have been quantified for some vertebrate species but are yet to be quantified for insects. Investigating the road‐effect zone for insects will provide a better understanding of how roads impact ecosystems, which is particularly important given the role insects play as pollinators, predators, and prey for other species. We quantified the road‐effect zone for nocturnal flying insects along three major freeways in agricultural landscapes in southeast Australia. We collected insects using light traps at six points along 2‐km transects perpendicular to each highway (*n* = 17). We sorted the samples into order, and dried and weighed each order to obtain a measure of dry biomass. Using regression models within a Bayesian framework of inference, we estimated the change in biomass of each order with distance from the road, while accounting for environmental variables such as temperature, moon phase, and vegetation structure. The biomass of nine of the ten orders sampled did not change with distance from the freeway. Orthoptera (i.e., grasshoppers and crickets) was the only order whose biomass increased with distance from the freeway. From our findings, we suggest that the impacts of roads on insects are unlikely extending into the surrounding landscape over a distance of 2 km. Therefore, if there are impacts of roads on insects, these are more likely to be concentrated at the road itself, or on finer taxonomic scales such as family or genus level.

## 1 INTRODUCTION

Insect populations are in decline globally (Baxter‐Gilbert, Riley, Neufeld, Litzgus, & Lesbarreres, 2015; Hallmann et al., 2017; Potts et al., 2010). For example, the seasonal biomass of flying insects in Germany has declined 76% in <30 years (Hallmann et al., 2017). As a fundamental part of the ecosystem, changes to insect community assemblages and abundance can be detrimental to the overall system (e.g., Dirzo et al., 2014; Yang & Gratton, 2014). Insects provide many ecosystem services such as pollination and nutrient recycling (e.g., Dirzo et al., 2014; Yang & Gratton, 2014) and are the primary food resource for many mammals, birds, reptiles, amphibians, and carnivorous plants (e.g., Scudder, 2009). Thus, a loss of insects can result in ecosystem crashes and cascades through the different levels of the food web (e.g., Scudder, 2009; Dirzo et al., 2014). The decline of insect populations is often thought to be driven by the loss and degradation of habitat (e.g., Nilsson, Franzen, & Jonsson, 2008; Winfree, Aguilar, Vazquez, LeBuhn, & Aizen, 2009). However, one pervasive threat that is understudied is the impact of roads and traffic on insect biomass and distribution.

There are more than 64 million kilometers of roads fragmenting landscapes across the globe. Roads can have detrimental impacts on invertebrate species, including barrier effects (Knapp et al., 2013; Koivula & Vermeulen, 2005), and road mortality (Baxter‐Gilbert et al., 2015; Keilsohn, Narango, & Tallamy, 2018; Martin et al., 2018; Rao & Girish, 2007; Seibert & Conover, 1991). However, the ecological impacts of roads are not restricted to the site of the road itself, and they often extend into the surrounding landscape, over a distance known as a “road‐effect zone” (Forman & Alexander, 1998). The size of the road‐effect zones depends on the characteristics of the road and landscape, and can extend across several kilometers. For example, roads potentially impact 15%–20% of the landscape of the USA, despite only covering 1% of its land area (Forman & Alexander, 1998). Habitat degradation from road impacts such as traffic noise (e.g., Parris & Schneider, 2009; McClure, Ware, Carlisle, Kaltenecker, & Barber, 2013) and changes in vegetation structure (e.g., Berthinussen & Altringham, 2012) can reduce the suitability of roadside habitats, thus increasing the area of land affected by the road. Road‐effect zones have been quantified for a number of vertebrate groups including birds, amphibians, and mammals (For review please see: Benitez‐Lopez, Alkemade, & Verweij, 2010). However, a road‐effect zone has not been investigated for insect populations.

Quantifying a road‐effect zone for insects is important to understand the broader impacts of roads on wildlife populations living in the surrounding landscape (Reck & van der Ree, 2015). In addition to contributing to our knowledge of potential drivers of insect population declines, understanding the road‐effect zone for insects may also improve our knowledge of the cause of road‐effects for insectivores (Reck & van der Ree, 2015). For example, road‐effect zones have been quantified for insectivorous bats and amphibians (e.g., Eigenbrod, Hecnar, & Fahrig, 2009; Berthinussen & Altringham, 2012), and it is possible that this could be driven by a corresponding road‐effect zone affecting their primary food source—insects. Identifying the cause of the road‐effect zone is important for wildlife managers, because mitigation strategies can be costly and if they are ill‐suited to mitigate the true cause of the impact, they are unlikely to be effective. Therefore, quantifying the road‐effect zone for insects may help managers to mitigate the impacts of roads on insects and insectivores alike and create targeted mitigation strategies aimed to reduce the size and severity of the road‐effect zone.

In this study, we quantify the road‐effect zone for nocturnal, flying insects, which are the primary prey for nocturnal insectivores such as bats and some species of frogs and birds. We compared the biomass of ten insect orders with increasing distance from three major freeways in southeast Australia. Findings from this study will give insight into the changes of insect composition in the habitat surrounding roads, which in turn may provide insight into the intricate relationship between insects and insectivores. As an integral part of the natural environment, understanding the causes for changes to insect populations may be essential to maintaining sustainable, functioning ecosystems.

## 2 MATERIAL AND METHODS

### 2.1 Study area

**Figure 1:**
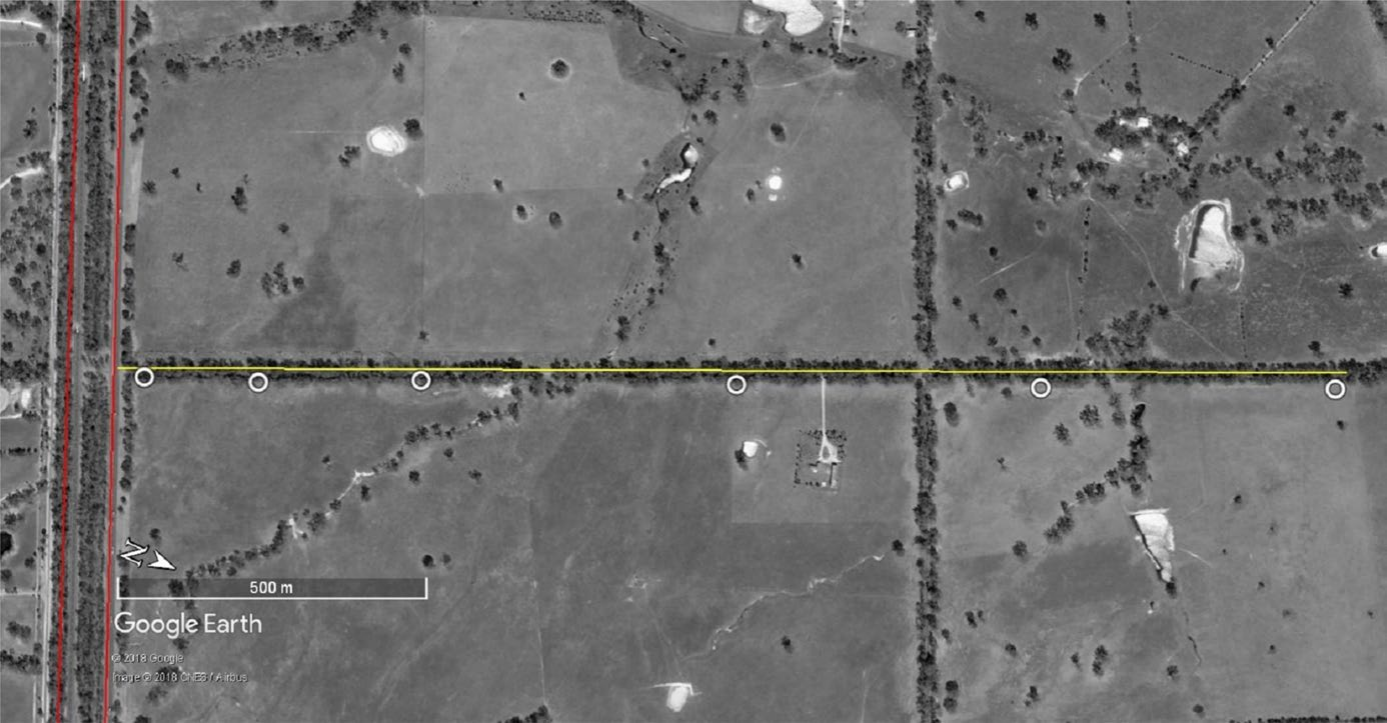
Example of a transect from an aerial view, showing the dual carriage freeway in red on the left of the frame and the transect in yellow. The white circles show the light trap placements at 25, 250, 500, 1,000, 1,500, and 2,000 m from the freeway. Photograph from Google Earth 2017

This study was conducted during the 2014/2015 Australian summer, from December to February, in central Victoria, Australia. Data were collected along three major freeways: the Hume Freeway, the Goulburn Valley Freeway, and the Calder Freeway. These freeways are four‐lane divided motorways—two lanes in each direction. Each carriageway is approximately 12 m wide, and they are separated by a vegetated median that ranged 5–20 m in width. Within the study area, the maximum speed limit of these freeways is 110 km/h. The annual average daily traffic volume (in one direction) ranges from 5,800 to 6,300 vehicles/day (average 6,140 vehicles/day) along the Hume Freeway, from 3,700 to 4,800 vehicles/day (average 4,460 vehicles/day) along the Goulburn Valley Freeway, and from 5,500 to 9,100 vehicles/day (average 6,720 vehicles/day) along the Calder Freeway (VicRoads, 2015). The landscape surrounding the freeways is predominantly farmland with patches of native vegetation, consisting mainly of heathy dry forest, with some grassy woodlands and box ironbark forest (Costermans, 2006). We collected insect samples along 17 small, single‐lane roads that meet the freeways perpendicularly (henceforth referred to as “transects”). The traffic on transects was low, on average fewer than 100 vehicles per day, and we did not expect this traffic to have an influence on insect abundance or diversity along the transects. All transects were tree‐lined on both sides of the road, and adjacent to farm paddocks and scattered residences (Figure 1; further information on each transect is provided in the Supporting Information Table S1 and site‐level images are provided in Supporting Information Figure S1). The freeways and the transects were all unlit. Water bodies near the transects were dry at the time of the data collection. The transects were distributed along the three freeways: Hume Freeway (*n* = 7), Calder Freeway (*n* = 5), and Goulburn Valley Freeway (*n* = 5).

### 2.2 Data collection

We collected photosensitive, nocturnal flying insects using light traps. Light traps consisted of a white 10 L bucket containing one UV light tube and one white light tube with a container of ethanol at the bottom into which insects were funnelled (following Lumsden & Bennett, 2005). The light traps were powered by two 12 V batteries, which were programmed to turn on at sunset and off at sunrise, thereby collecting samples all night. Light traps were hung on an outer branch of a tree, approximately 2 m from the ground, at six distances from the freeway: 25, 250, 500, 1,000, 1,500, and 2,000 m. Sampling distances were chosen taking into consideration the catchment size of the light traps (approximately a radius of 50 m; Patrick, 2016). Insects were sampled for two consecutive nights at each transect, and insects were collected from each trap after each night. Due to a lack of a suitable tree (i.e., with an accessible limb that could bear the weight of the light trap), and equipment malfunctions, we were not able to collect a sample from each distance every night on all transects. We obtained a total of 151 nightly samples during the study (25 m, *n* = 23; 250 m, *n* = 27; 500 m, *n* = 27; 1,000 m, *n* = 26; 1,500 m, *n* = 28; 2,000 m, *n* = 20; maximum potential number of collections per distance was 34, with 204 sample collections overall).

At each point along the transect, we also conducted habitat assessments within a 10 m radius of the trunk of each tree on which a light trap was hung. We recorded the species and size of all trees and shrubs present, and the canopy cover (visually assessed and assigned a percentage of cover). Moon phase was recorded from Museum Victoria data (https://museumvictoria.com.au/planetarium/discoverycentre/moon-phases/). Daily minimum temperatures were obtained from the Australian Government, Bureau of Meteorology (https://www.bom.gov.au/climate/data/), and typically reflect overnight temperatures.

Insects from each trap were sorted to order, and then dried at 60°C in a conventional oven. During drying, samples were weighed every hour until the mass of the sample was consistent for two consecutive readings. Ten insect orders were present in the samples: Coleoptera (e.g., beetles), Diptera (e.g., flies), Hemiptera (e.g., true bugs), Hymenoptera (e.g., wasps and bees), Isoptera (e.g., termites), Lepidoptera (e.g., moths and butterflies), Neuroptera (e.g., lacewings), Odonata (e.g., dragonflies and damselflies), Orthopotera (e.g., crickets and grasshoppers), and Trichoptera (e.g., caddisflies). On average, 8.94 g of total dry insect biomass was collected per light trap per night (range: 0–69.67 g; median 3.65 g) over the 151 trap nights of sampling (Table 1).

**Table 1:** Mean, range, and median of dry biomass per night for all insect orders combined, and for each order separately, and the percentage of trap nights they were recorded

Order	Mean dry biomass (g)	Dry biomass range (g)	Median dry biomass (g)	Percent of trap nights present
Coleoptera	5.53	0.00–68.09	1.81	92
Diptera	0.04	0.00–0.59	0.02	81
Hemiptera	0.03	0.00–0.58	0.00	32
Hymenoptera	0.10	0.00–2.22	0.02	68
Isoptera	0.01	0.00–0.55	0.00	26
Lepidoptera	2.98	0.00–40.39	1.37	96
Neuroptera	0.02	0.00–0.62	0.00	53
Odonata	0.00	0.00–0.18	0.00	9
Orthoptera	0.20	0.00–2.27	0.00	48
Trichoptera	0.01	0.00–0.25	0.00	38
All insects combined	8.94	0.00–69.67	3.65	100

### 2.3 Statistical analysis

Order richness (i.e., the presence of each order) did not change with distance from the freeway (Spearman's rank correlation: rho = 0.02, *S* = 561,750, *p* = 0.80), so the primary analysis is focused on the change in biomass of each order with distance from the freeway. To compare the change in biomass with distance from the freeway, we fitted linear regression models using the log of biomass as a response (biomass ranged from 0 to 68.09 g across the orders; mean, median and range of biomasses for each order are provided in Table 1). For each data point *i* (each trap, per night):

$$\text{log}\left(B_{i}\right)\;\;N\left(\mu_{i},\;\sigma^{2}\right)$$

$$\mu_{i}=\;\beta_{0}+\beta_{1}D_{i}+\beta_{2}T_{i}+\beta_{3}M_{i}+\beta_{4}S_{i}+\beta_{5}N_{i}+\beta_{6}C_{i}+\beta_{7}H_{i}+\varepsilon_{x\left(i\right)}$$

where μ*_i_* was the mean log biomass (*B_i_*). *D* represents the distance from the freeway at which the light trap was placed along the transect. More specifically, we used *D* = log(distance+1) to reflect the diminishing strength with distance, as expected from a potential road‐effect. We added 1 to the distance so that we could estimate the potential biomass at 0 m from the freeway. We compared this model with one using a linear relationship with distance (standardized) and found that the log approach generally provided better fitting models (lower or similar DIC values). To account for nightly variation in environmental conditions, we included daily minimum temperature (*T*) and moon phase (*M*, 3 categories: new moon, full moon, first/last quarter). New moon was used as the reference; more nocturnal flying insects tend to be trapped during moonless nights, compared to full moon or partial moon nights (Lawer & Darkoh, 2016; Nowinszky, Puskas, & Kuti, 2010). To account for site‐level variation, we included several measures of vegetation structure, within a 10 m radius of the light trap: richness of tree and shrub species (number of different tree and shrub species in a 10 m radius, *S*); number of large trees (diameter > 30 cm at breast height, *N*); and canopy cover (*C*) (Ober & Hayes, 2008). *H* represents a fixed‐effect of freeway (three categories: Hume Freeway, Goulburn Valley Freeway, and Calder Freeway; Calder Freeway was used as reference) to account for potential differences in insect abundance that may result on the local scale among the freeways. Finally, we included a random‐effect term for the transect, $\varepsilon_{x\left(i\right)}$ to account for local baseline differences in insect abundance. All continuous covariates other than distance (i.e., minimum daily temperature, number of trees >30 cm diameter at breast height, trees species richness, and canopy cover) were standardized around the mean with variance 1. The intercept (*β*_0_) represents the baseline: the expected log biomass at mean log(distance + 1), mean temperature, reference moon phase (new moon), mean tree species richness, mean number of large trees, mean canopy cover, and reference freeway (Calder Freeway).

All model fitting was conducted within a Bayesian framework of inference using Markov Chain Monte Carlo (MCMC) sampling, by calling JAGS 4.1.0 (Plummer, 2003) from R (v3.3.2; R Core Team, 2016) using package R2jags (Su & Yajima, 2015). We used uninformative priors for all parameters: uniform distributions *U*(−10,10) for all regression coefficients. We fitted this model for total biomass (biomass of all orders combined) and for each order separately. We ran three MCMC chains for each parameter, keeping 100,000 iterations after discarding a burn‐in of 50,000. Convergence was assessed by visual inspection of the chains and using the statistic R‐hat (assuming no evidence of lack of convergence for values below 1.01).

## 3 RESULTS

**Figure 2:**
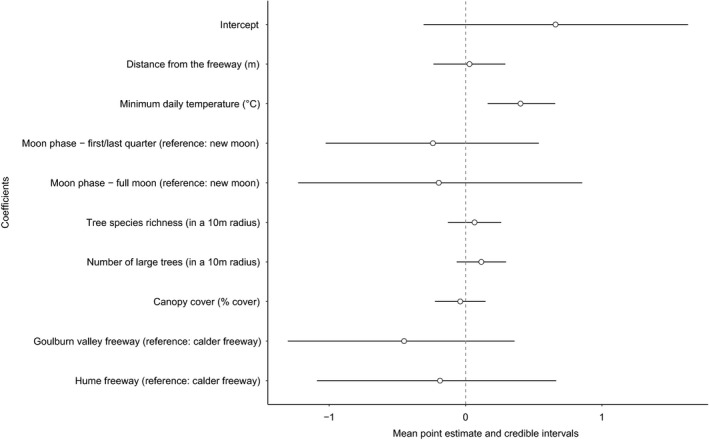
Point estimate of the mean and 95% credible intervals (bars) for the regression coefficients included in the model for the combined insect biomass. Credible intervals overlapping 0 (dotted line) indicate that the corresponding effect is not significant at the 5% level

**Figure 3:**
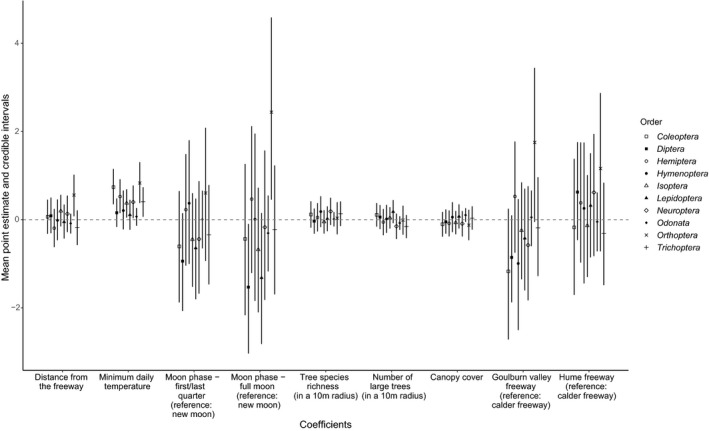
Point estimates of the mean and 95% credible intervals (bars) for the regression coefficients included in the model, reflecting the output of the models using each individual order's biomass as a response. Credible intervals overlapping 0 (dotted line) indicate that corresponding effect is not significant at the 5% level. Distance from the freeway was only a significant predictor of Orthoptera biomass

Distance from the freeway was not a significant predictor of the biomass of all insects combined (Figure 2) or for any order except Orthoptera (Figure 3) (graphs of estimated biomass at each distance, for each order, are provided in the Supporting Information, Figure S2). Minimum daily temperature was a positive predictor of biomass; this relationship was significant for all insect orders combined, and for Coleoptera, Hemiptera, Isoptera, Neuroptera, Orthoptera, and Trichoptera individually, but not for Diptera, Hymenoptera, Lepidoptera, and Odonata (Figure 3). Biomass did not change significantly between moon phases for most orders; however, Dipteran biomass was significantly lower during a full moon compared to a new moon and Orthopteran biomass was significantly higher during a full moon compared to a new moon (Figure 3). Consistent with the study design to keep these factors constant, the species richness of trees and shrubs, the number of large trees, and canopy cover were not significant predictors of insect biomass in any model. Finally, average biomass per trap did not differ significantly among freeways (Figure 3).

## 4 DISCUSSION

Roads and road networks can have detrimental impacts on wildlife populations. In the present study, we aimed to identify a road‐effect zone for nocturnal, flying insects in the agricultural landscapes of Victoria, Australia. We determined that the richness of orders (i.e., the presence of each order) did not change with distance from the freeway. We also determined that biomass for nine out of ten insect orders did not change with distance from the freeway. The order Orthoptera, which includes crickets and grasshoppers, was the only order whose biomass increased with distance from the freeway. This may be due to the lower quality of habitat in the freeway verge. Orthopterans will use long grassy vegetation as a refuge (Humbert, Ghazoul, Richner, & Walter, 2012); however, the freeway verges in this study area tended to have the grassy vegetation mowed. Additionally, as an order that relies on acoustic communication, Orthopterans may have not been found close to the freeway because traffic noise and vibrations can hinder their ability to communicate (Morley, Jones, & Radford, 2014). Overall, our results suggest that the impacts of roads on most nocturnal flying insects do not extend into the surrounding habitat.

Our results do not support the hypothesis that a road‐effect zone for nocturnal insectivores could be explained by the lack of availability of insect prey. The only exception would be for species that feed primarily on Orthoptera. The higher‐than‐expected biomass of insects near roads may be from the lack of predation pressure from species that are vulnerable to road‐effect zones, such as bats (Berthinussen & Altringham, 2012) and frogs (Eigenbrod et al., 2009), creating refuge for insects in roadside habitat. This type of relationship has been observed in white‐footed mice (*Peromyscus leucopus*), where the road verge acts as a relatively predator‐free refuge and mice abundance increases with proximity to the road (Rytwinski & Fahrig, 2007). However, the way in which roads alter community composition and interspecies interactions is generally understudied, making generalizations across systems and species difficult, leaving us with only a piecemeal understanding of effects of roads at the community or ecosystem level.

The mechanisms driving road‐effect zones can be complex, and it is important that studies aim to identify the causal factors and control for potential confounding variables as much as possible. For example, we controlled for the effect of vegetation structure on insect abundance/diversity and risk of road mortality (Keilsohn et al., 2018; Ober & Hayes, 2008), by selecting transects that were as similar in vegetation structure as possible (along transects, among transects, and among freeways). Thus, as expected, canopy cover, number of large trees, and tree and shrub species richness did not have a significant influence on the biomass of insects. However, other factors that could have also influenced the results were harder to control for. For example, daily minimum temperature, which fluctuated from night to night, had a positive effect on insect biomass (i.e., biomass increased with increasing temperature). Thus, in order to fully understand and isolate the impact of the road, it is important to understand the influence of other confounding variables.

Further studies are required to assess the generality of our results under different landscape contexts, spatial scales, and taxonomic levels. For example, while artificial roadside lighting was not present in our study, it is likely to be an important consideration for photosensitive species along lit roads. Also, our study focussed on detecting a road‐effect zone at a relatively large spatial scale (up to 2 km) and did not explore the potential for fine‐scale effects immediately adjacent to the freeway (e.g., within the first 25 m). Additionally, the transects in this study were low‐traffic roads themselves, which could have confounded our results, thus evaluating the change in insect biomass along transects that are not defined paths would also improve our knowledge of the system. Finally, we measured the response at the level of select insect orders. Widening the scope of orders that were collected (e.g., diurnal or terrestrial orders) and identifying insects to a finer taxonomic level may uncover more nuanced responses, and further our understanding on community‐level processes such as pollinator interactions and predator–prey interactions.

Although we did not detect a road‐effect zone for the majority of the orders studied, we should not disregard the other impacts that roads have on insects (Muñoz, Torres, & Megias, 2015). Insects are subjected to road mortality (Rao & Girish, 2007; Seibert & Conover, 1991), barrier impacts (Baxter‐Gilbert et al., 2015; Knapp et al., 2013; Koivula & Vermeulen, 2005), and habitat loss due to mowing (Humbert et al., 2012). As a vital part of the ecosystem, a greater understanding of the impact of roads on insect populations is important to maintaining a functioning ecosystem.

Ultimately, there is much left to understand about the road‐effect zone. Future research should quantify the size of the road‐effect zone for different taxa, and also target the causal mechanism of the road‐effect zone. It is important to see how the road‐effect zone changes with the size of roads and the volume of traffic traversing the road (e.g., Martin et al., 2018), the features of the road such as finishing (i.e., asphalt road, dirt road) and lighting, the type of habitat the roads transect, and the current state of a population's success (i.e., if they are already in decline or if they are thriving in the area), in order to target appropriate mitigation measures. The more information we have, the better we can anticipate and mitigate the broad‐scale, ecosystem‐level impacts of roads.

## CONFLICT OF INTEREST

None declared.

## AUTHORS’ CONTRIBUTIONS

MB, RvdR, and LL conceived the ideas and designed methodology; MB collected the data; MB, KS, and JLM analyzed the data; MB, KS, JLM, LL, and RvdR interpreted the results; MB led the writing of the manuscript. All authors contributed critically to the drafts and gave final approval for publication.

## DATA ACCESSIBILITY

Data accessible at https://doi.org/10.6084/m9.figshare.6791903.v1.
