# Supplementary material for: Little evidence of a road‐effect zone for nocturnal, flying insects
Source: Ecol Evol. 2018 Dec 27;9(1):65–72. doi: 10.1002/ece3.4609 (PMC6342180; doi:10.1002/ece3.4609)
Supplement: Supplementary file 1 [file ECE3-9-65-s001.docx]

Bhardwaj et al. Little Evidence of a Road Effect Zone for Nocturnal, Flying Insects: Supplementary Information

Table S1. Descriptions of each transect, including the width of each transect, the boundary width (encompassing the road verges and the road entirely), the average road verge width along the length of the transect, the type of finishing, and the number of houses adjacent to the transect, and within 5 km of the freeway. These variables are provided to indicate the type of roads and the type of traffic that would have been travelling along each transect.

| Transect | Freeway | Road Width (m) | Boundary Width (m) | Average Road Verge Width (m) | Road Finishing | Number of Houses within 5 km from the Freeway |
| --- | --- | --- | --- | --- | --- | --- |
| Aerodrome | Goulburn Valley Freeway | 4.5 | 19.6 | 8 | Dirt | 1 |
| Alexanderson | Hume Freeway | 9.6 | 34.2 | 14 | Gravel | 2 |
| Allendale | Calder Freeway | 6.2 | 6.6 | 6 | Bitumen | 1 |
| Balmattum-Siding | Hume Freeway | 4.6 | 21.6 | 9 | Bitumen | 7 |
| Benalla-Warranbayne | Hume Freeway | 11.0 | 22.3 | 7 | Bitumen | 18 |
| Boho | Hume Freeway | 10.5 | 20.2 | 5 | Bitumen | 5 |
| Carters | Hume Freeway | 8.2 | 30.7 | 12 | Gravel | 15 |
| Cemetery | Goulburn Valley Freeway | 7.0 | 19.9 | 8 | Dirt | 11 |
| Gerrards | Goulburn Valley Freeway | 10.2 | 32.0 | 13 | Gravel | 3 |
| Hokins | Calder Freeway | 6.8 | 21.5 | 8 | Bitumen | 6 |
| Manglore | Goulburn Valley Freeway | 7.5 | 20.3 | 7 | Dirt | 3 |
| Owens | Calder Freeway | 4.5 | 20.0 | 8 | Dirt | 3 |
| Peck | Hume Freeway | 4.7 | 18.1 | 8 | Dirt | 1 |
| Pollards | Calder Freeway | 6.8 | 20.3 | 6 | Gravel | 21 |
| Potts | Calder Freeway | 5.6 | 21.3 | 7 | Gravel | 14 |
| Roachs | Hume Freeway | 4.3 | 19.0 | 7 | Dirt | 2 |
| Strays | Goulburn Valley Freeway | 4.8 | 19.3 | 7 | Dirt | 0 |


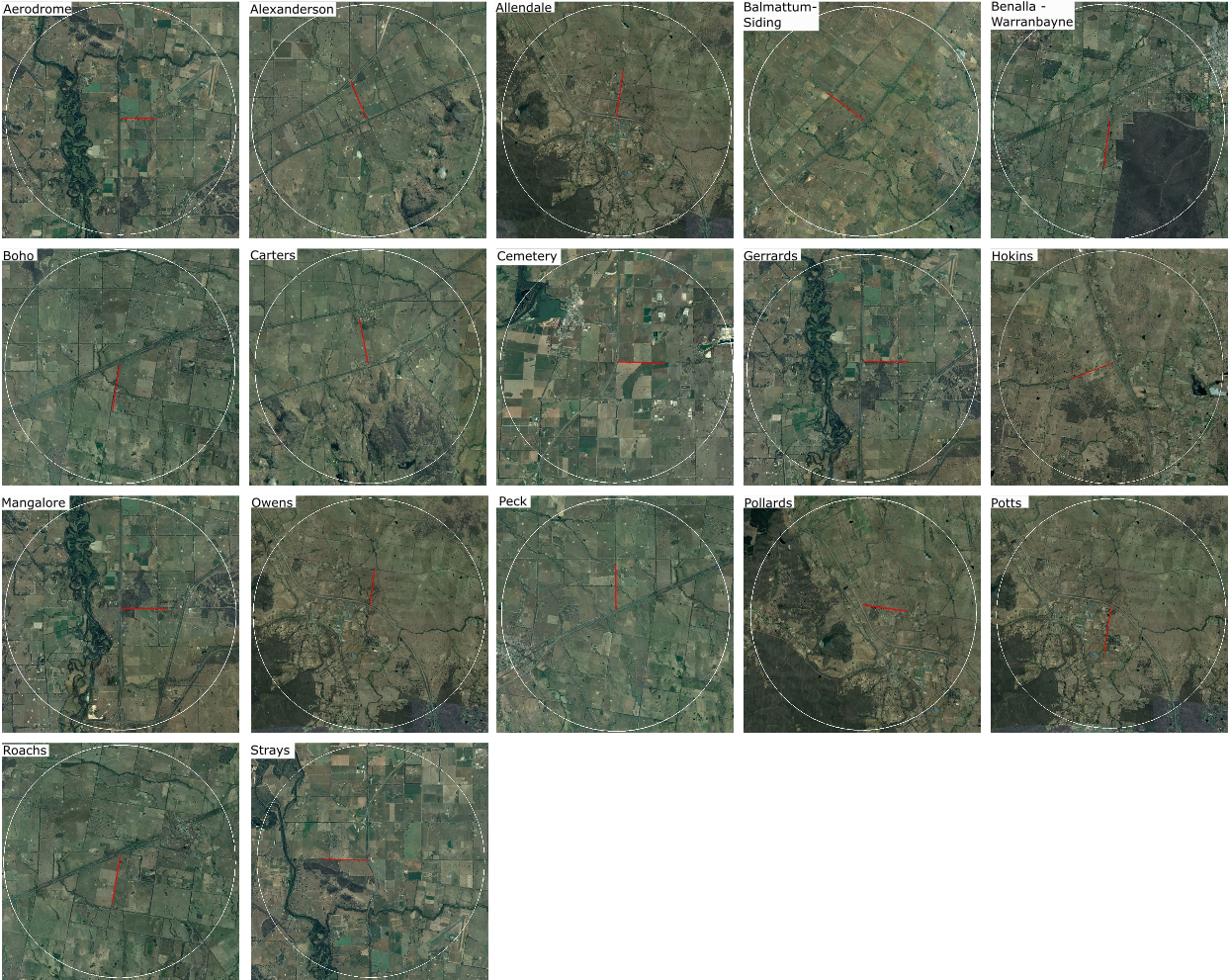


Fig S1. The 17 study area showing a 5 km radius (white circle) around the intersection where the transects meet the highway. The red lines indicate the 2 km length of the transect. Each image points north.


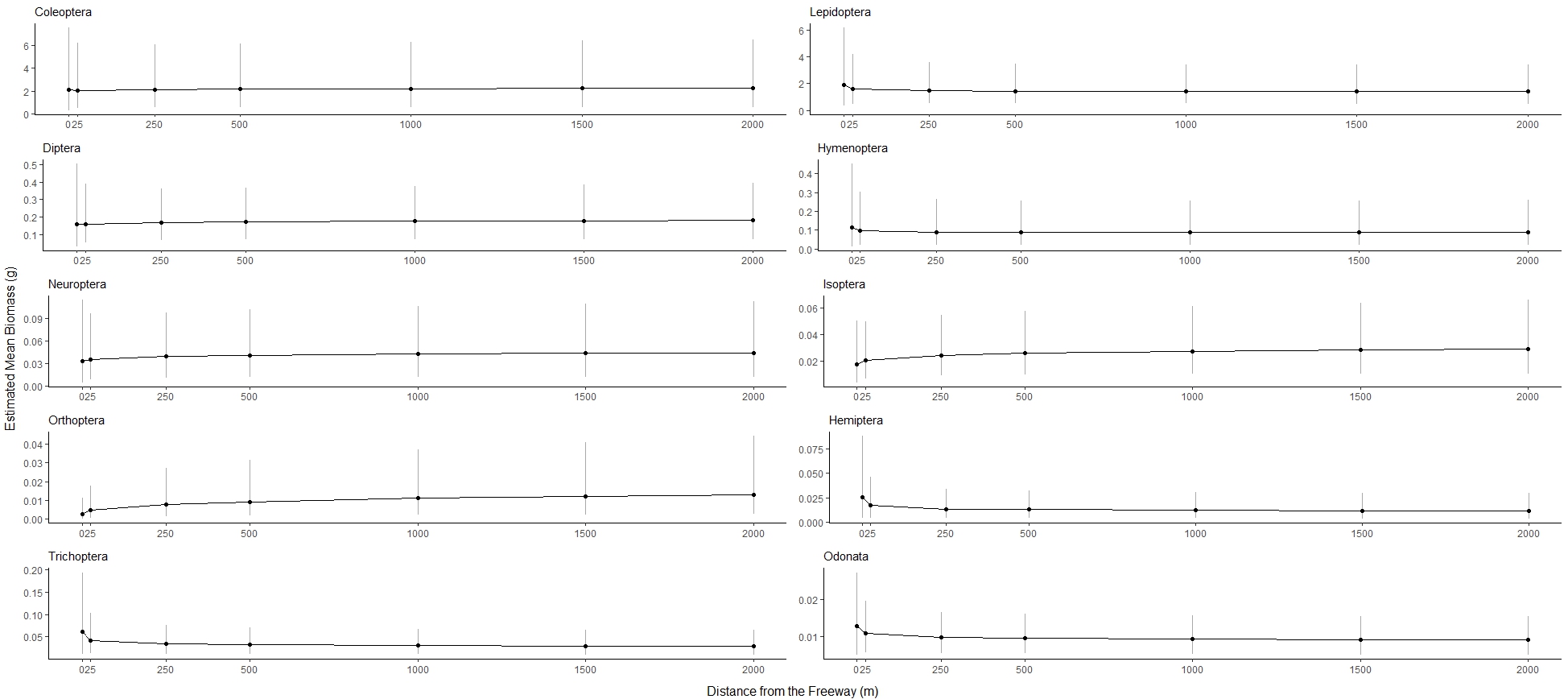


Figure S2. Estimated biomass (error bars show 95% credible intervals) of each order, based on their individual models. Biomass was estimated for each distance from the road that was monitored, and extrapolated for 0 m. The orders are arranged in descending order of upper y-axis value, thus arranged from those that had the highest potential biomass to the lowest. Note that the y-axis differs on each graph, to allow for clear visualization of all trends.
